# Fees for Dental Operations Reduced

**Published:** 1852-04

**Authors:** 


***Fees for Dental Operations Reduced.***—The use of unprofessional means to obtain practice is bad enough in an ignoramus, but when we see a man whose opportunities for obtaining information have been ample, descending to the trickery of charlatanism to secure practice, we take it for granted he is destitute of one very important requsite to a good practitioner, namely, moral honesty. Indeed, a man who will do this, should be held in utter contempt by every respectable member of the profession. These remarks have been elicited by an advertisement with the above *attractive* heading, recently sent to us by a friend in Virginia, of a dentist, who proposes to perform operations on the teeth, at prices, which, if faithfully executed, with constant employment, would leave him about three dollars out of pocket at the end of every day.

